# Income, Geography, and Etiology as Factors Associated with Valve Replacement Outcomes: A Nationwide Cohort of 63,426 Patients in a Middle-Income Country

**DOI:** 10.5334/gh.1581

**Published:** 2026-08-25

**Authors:** Leonardo Dexheimer da Silva, João Luís Freitas, Felipe Dircêu Dantas Leite Pessôa, Elinthon Tavares Veronese, Carlos Manuel de Almeida Brandão, Roney O. Sampaio, Guilherme Sobreira Spina, Flávio Tarasoutchi

**Affiliations:** 1 University of São Paulo Medical School (FMUSP), São Paulo, Brazil; 2 Heart Institute of University of São Paulo Medical School (INCOR –FMUSP), São Paulo, Brazil

**Keywords:** heart valve surgery, prosthetic valve replacement, rheumatic heart disease, low- and middle-income countries, health inequity

## Abstract

**Background:**

Heart valve disease is a growing cause of mortality and disability worldwide, disproportionately burdening low- and middle-income countries (LMICs), where rheumatic heart disease remains a major etiology and cardiac surgical capacity covers less than 2% of the estimated need. Population-level outcome data after valve surgery in LMICs are virtually absent. We leveraged Brazil’s universal health system to quantify how income, geography, and disease etiology are associated with survival after valve replacement surgery.

**Methods:**

Five Brazilian public health databases covering outpatient care (SIA), hospital admissions (SIH), mortality records (SIM), the cancer registry (RHC), and a social vulnerability index (IVS Atlas) were linked to a single individual-level longitudinal record, with a proof-of-life algorithm correcting right-censoring bias. All patients undergoing valve replacement surgery between January 2015 and December 2024 were included.

**Results:**

Among 63,426 patients (mean age 56.9 ± 15.2 years; median follow-up 1,023 days), the in-hospital mortality rate was 8.6% and the five-year mortality was 30.1%. Income and geography were significantly associated with survival: the highest income quartile had significantly lower mortality than the lowest quartile in the adjusted Cox model (HR 0.82; 95% CI 0.76–0.89; p < 0.001), and in-hospital mortality exceeded 25% in low-volume municipalities in the North region. Valve lesion subtype predicted outcomes differentially across time horizons, and reoperation was associated with worse survival (HR 1.48; 95% CI 1.37–1.58; p < 0.001).

**Conclusion:**

Income, geography, and valve-specific risk factors are important factors associated with survival after valve surgery in Brazil’s public health system, reflecting structural conditions shared across LMICs. The linkage framework offers a scalable model for cardiovascular outcome surveillance in resource-constrained settings.

**Lay Summary:**

Each year, thousands of Brazilians undergo heart valve surgery through the public health system. Using data from over 63,000 patients treated between 2015 and 2024, we found that survival after valve surgery in Brazil is substantially worse than in wealthier countries and that outcomes differ markedly depending on which valve is affected and why. Patients who required a second surgery fared worse, and those living in regions with fewer surgical centers faced higher mortality. These findings highlight critical gaps in cardiac surgical care, which can guide resource allocation and policy in Brazil and similar settings.

## Graphical Abstract

**Figure:**
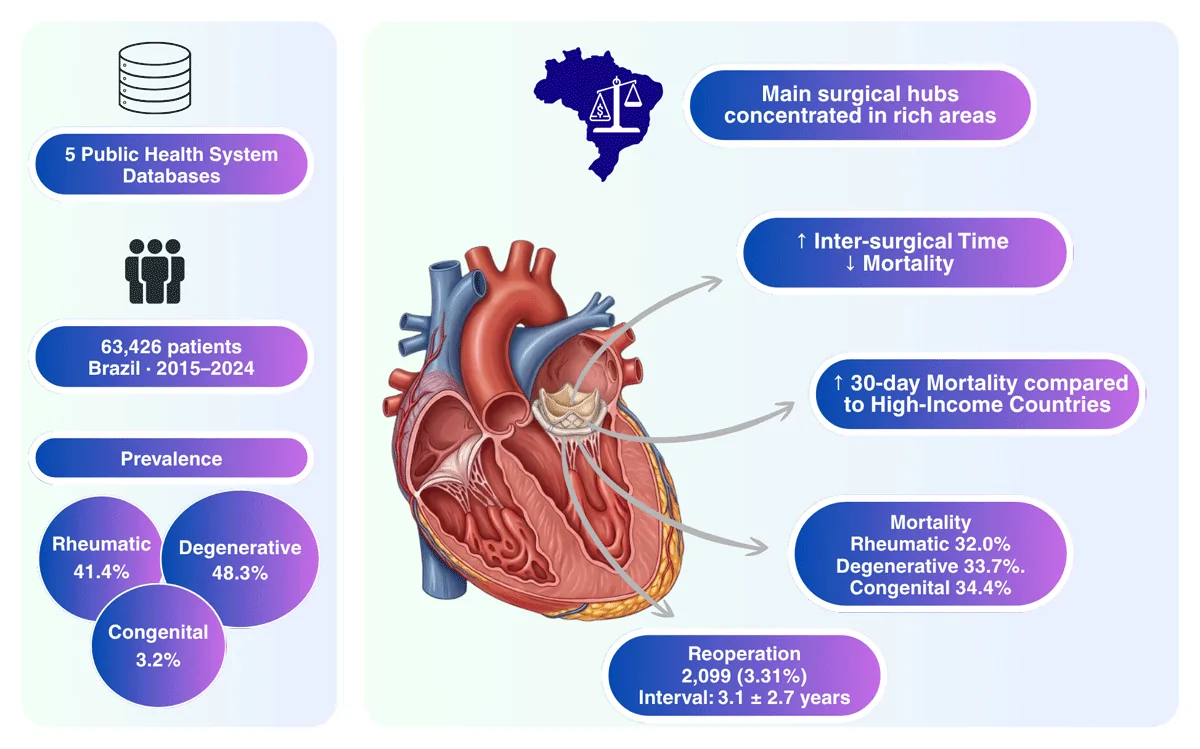


## 1. Introduction

Valvular heart disease is a growing cause of mortality and disability worldwide. Data from the Global Burden of Disease Study 2021 document a consistent increase in global prevalence over the past three decades, while age-standardized disability-adjusted life years declined between 1991 and 2021 (1). In high-income countries, degenerative etiologies associated with aging predominate. In low- and middle-income countries (LMICs), rheumatic heart disease remains the dominant cause of valvular disease, closely linked to poverty and household overcrowding (2,3,4). An estimated 33.4 million people were living with rheumatic heart disease in 2015, accounting for approximately 319,400 annual deaths globally, a burden disproportionately concentrated in LMICs (5). Simultaneously, more than six billion people reside in regions where cardiac surgical capacity covers less than 2% of the estimated need, with installed capacity ranging from 0.5 to 7 operations per million people (6,7,8).

In Brazil, approximately 30,000 new cases of acute rheumatic fever occur annually, and roughly one third of all cardiovascular surgeries are attributable to rheumatic sequelae (8). The BYPASS Registry, a prospective multicenter Brazilian registry comprising 920 patients from 17 centers, reported an in-hospital mortality rate of 7.3%, compared with 2.2% in the EURObservational VHD II survey (9,11). Marked regional disparities compound this scenario: cardiovascular surgeon density ranges from 1.75 per 100,000 people in São Paulo to 0.16 per 100,000 in Roraima (10).

Population-level, long-term outcome data after valve surgery in LMICs are scarce. Existing registries are predominantly single-center or voluntary multicenter cohorts, and no study has used national administrative databases to reconstruct individual trajectories at scale. Although administrative linkage for cardiac surgical surveillance is established in high-income countries, such as through the EURObservational Research Programme (11), it remains largely unexplored in settings with a high burden of rheumatic disease. Brazil, as the world’s largest single-payer health system, provides both the epidemiological context and the data infrastructure to quantify the structural factors associated with valve surgery outcomes at a population level. The patterns of inequity identified here, across income, geography, and disease etiology, are likely to reflect conditions common to LMICs where similar barriers to surgical access persist.

This study aimed to: i) assess the associations between etiology, socioeconomic profile, and post-operative survival outcomes across the perioperative, intermediate, and late follow-up periods after prosthetic valve replacement in Brazil’s Unified Health System (SUS), using a nationwide retrospective cohort linking five administrative databases and ii) to propose a replicable surveillance framework for cardiovascular outcomes in resource-constrained settings.

## 2. Methods

### 2.1 Study design and data sources

This retrospective nationwide cohort study used individual-level administrative data from the SUS, integrating five databases: the Ambulatory Information System (SIA), the Hospital Information System (SIH), the Mortality Information System (SIM), the Hospital Cancer Registry (RHC), and Atlas of Social Vulnerability (IVS) of the Institute for Applied Economic Research (IPEA). Records were linked using encrypted patient identifiers and analyzed in de-identified form. The SIA provided outpatient encounters, high-complexity procedures, and medication dispensing, forming the basis for longitudinal follow-up. The SIH captured hospital admissions and surgical procedures. The SIM was used for mortality ascertainment. Residential ZIP codes were geocoded and mapped to Human Development Units (UDH) to assign the Municipal Human Development Index (IDHM) and its income subcomponent.

### 2.2 Cohort definition

Identified in SIH using the procedure code 0406010692, all patients undergoing at least one valve replacement surgery within the SUS between January 2015 and December 2024 were included. The index date was defined as the first recorded procedure. Etiology was classified using ICD-10 codes recorded in SIA as rheumatic (I05–I09), degenerative (I34–I37), or congenital (Q22–Q23); prosthetic valve status was identified using codes Z95.2–Z95.3. Administrative histories were reconstructed from the index date until death or end of follow-up (December 31, 2024).

### 2.3 Record linkage and longitudinal reconstruction

Records were linked using a composite identifier derived from encrypted demographic and administrative variables, including date of birth, sex, municipality code, National Health Card (CNS) number, and hospital identifiers. A hierarchical deterministic approach was applied in successive passes of decreasing stringency, and records with demographic inconsistencies were excluded. Linkage accuracy, assessed through internal consistency of recorded dates of death and longitudinal records, was approximately 90%.

### 2.4 Mortality ascertainment

All-cause mortality was ascertained through deterministic and probabilistic linkage to SIM using a cascading strategy that accounted for inter-municipal mobility. To reduce right-censoring bias from administrative loss to follow-up, a proof-of-life algorithm was applied: patients with any active SUS contact after the index date were considered alive through that date. Patients with no recorded death in SIH, SIM, or supplementary databases were considered alive at December 31, 2024.

### 2.5 Socioeconomic profiling

Residential ZIP codes were geocoded and assigned to UDH micro-regions. The income subcomponent of the IDHM was used to stratify patients into quartiles (Q1–Q4, lowest to highest income), providing a municipal-level proxy for socioeconomic context, access to care, and disease presentation stage.

### 2.6 Geographic and hospital-level analysis

Patient flows were reconstructed by linking the residential municipality to the hospital municipality of the index procedure. A directed referral network was constructed and weighted by PageRank centrality to identify dominant surgical hubs. In-hospital mortality rates were computed by municipality and mapped spatially to characterize the geographic distribution of surgical access and outcomes. Institutional surgical volume was assessed using the National Registry of Health Establishments (CNES) identifier linked to each patient’s index admission; hospitals were classified as high-volume (>200 cases/year) or low-volume (≤200 cases/year).

### 2.7 Statistical analysis

Continuous variables are reported as mean (SD) or median (IQR), and categorical variables as counts and proportions. Survival was estimated using the Kaplan–Meier method and compared with the log-rank test. A multivariable Cox proportional hazards model was pre-specified as the primary analysis for all-cause mortality, incorporating etiology, reoperation status, inter-surgical interval, age, sex, year of surgery, region, and income quartile as covariates, and provided the estimate of the association between income and survival. Landmark analyses at 30 days and five years stratified this same Cox framework into perioperative, intermediate, and late survival phases, rather than constituting a separate analysis; the 30-day cutoff was chosen to align with conventions used in comparator registries (12,13,14), while the five-year cutoff was chosen to separate early perioperative and prosthesis-related risk from late structural valve deterioration. Logistic regression was used to assess the secondary question of the association between reoperation and in-hospital mortality, with results expressed as odds ratios (ORs) and 95% confidence intervals. In the rheumatic subgroup, valvulopathy subtypes were compared using mitral stenosis as reference. All analyses were conducted in Python 3.11 using lifelines, statsmodels, and SciPy, with a two-sided alpha of 0.05.

## 3. Results

A total of 63,426 patients were retained after the linkage of 65,654 SIH surgical records with outpatient and administrative databases. Mortality ascertainment identified 5,451 in-hospital deaths, with 21,130 deaths recorded over complete follow-up (Table 2). A retrospective review of prior SIH records identified 977 patients with previous valve surgery.

### 3.1 Study population and patient characteristics

The cohort comprised degenerative (n = 30,620; 48.3%), rheumatic (n = 26,261; 41.4%), and congenital (n = 2,056; 3.2%) etiologies, with 4,489 (7.1%) classified as mixed or other. Mean age was 56.9 ± 15.2 years; degenerative patients were older (59.6 ± 14.5 years) and congenital patients were younger (51.4 ± 19.8 years). Males predominated in the degenerative (56.9%) and congenital (55.1%) groups, whereas rheumatic cases were more often female-dominated (56.4%). Comorbidities were infrequently recorded, including heart failure (5.8%), hypertension (4.4%), and diabetes (1.3%), consistent with known underreporting in administrative databases. Median follow-up was 1,023 days (IQR 277–2,183). Baseline characteristics are presented in Table 1.

**Table 1:** Baseline characteristics by valve disease etiology.


	RHEUMATIC	DEGENERATIVE	CONGENITAL	TOTAL	p-value

n	26261	30620	2056	63426	

Records per Patient	9.4 ± 34.7	11.9 ± 41.6	10.1 ± 38.4	10.7 ± 39.0	

Age (Mean ± SD)	54.5 ± 14.8	59.6 ± 14.5	51.4 ± 19.8	56.9 ± 15.2	**<0.001**

Male (%)	43.6%	56.9%	55.1%	51.4%	**<0.001**

Ethnicity					**<0.001**

White	9989 (38.0%)	17093 (55.8%)	891 (43.3%)	29824 (47.0%)	

Mixed Race (Pardo)	10469 (39.9%)	9043 (29.5%)	735 (35.7%)	21957 (34.6%)	

Black	996 (3.8%)	1185 (3.9%)	76 (3.7%)	2453 (3.9%)	

Yellow	420 (1.6%)	344 (1.1%)	26 (1.3%)	851 (1.3%)	

Unknown	4387 (16.7%)	2955 (9.7%)	328 (16.0%)	8341 (13.2%)	

Diabetes n(%)	295 (1.1%)	467 (1.5%)	24 (1.2%)	843 (1.3%)	**<0.001**

Hypertension n(%)	1129 (4.3%)	1449 (4.7%)	67 (3.3%)	2808 (4.4%)	**0.001**

Dyslipidemia n(%)	204 (0.8%)	396 (1.3%)	8 (0.4%)	644 (1.0%)	**<0.001**

Atrial Fibrillation n(%)	174 (0.7%)	193 (0.6%)	12 (0.6%)	424 (0.7%)	0.840

Renal Failure n(%)	136 (0.5%)	224 (0.7%)	11 (0.5%)	410 (0.6%)	**0.005**

Heart Failure n(%)	1065 (4.1%)	1485 (4.8%)	81 (3.9%)	3696 (5.8%)	**<0.001**

COPD n(%)	193 (0.7%)	328 (1.1%)	15 (0.7%)	571 (0.9%)	**0.036**

PT/INR Monitorization n(%)	695 (2.6%)	1069 (3.5%)	59 (2.9%)	1959 (3.1%)	**<0.001**

Length of Stay (Days)	12.7 ± 10.2	12.6 ± 9.9	14.7 ± 12.3	13.0 ± 10.6	**<0.001**

Follow-Up (Days) (Median (IQR))	1107.0 (306.0–2250.0)	999.0 (273.0–2171.2)	938.0 (212.8–2157.2)	1023.0 (277.0–2183.0)	**<0.001**

≥1-Year Follow-Up n(%)	19098 (72.7%)	21691 (70.8%)	1419 (69.0%)	45141 (71.2%)	

≥5-Year Follow-Up n(%)	9210 (35.1%)	10157 (33.2%)	646 (31.4%)	21249 (33.5%)	


### 3.2 Annual distribution of surgeries

Surgical volumes remained broadly stable from 2015 through 2019. In 2020, a decline of approximately 34% was observed, coinciding with the onset of the COVID-19 pandemic and the widespread suspension of elective procedures. A gradual recovery followed from 2021 onwards, with volumes approaching pre-pandemic levels by 2022 and continuing to rise through 2023. Annual in-hospital mortality rates followed a parallel pattern, declining gradually from 9.46% in 2015 to 7.80% in 2020, rising transiently to 9.03% in 2021 during the post-pandemic recovery phase, and then stabilizing between 7.95% and 8.34% from 2022 through 2024.

### 3.3 Geographic distribution of surgical volume, patient flows, and mortality

The geographic analysis revealed marked regional concentration in the provision of heart valve surgery across Brazil. Among the top 2,000 patient flow connections weighted by PageRank centrality, São Paulo was identified as the dominant referral hub (n = 3,395 patients received), followed by Recife (n = 1,695) and Teresina (n = 817). The network structure revealed dense inter-regional flows converging on these major centers, with municipalities in the North and Center-West relying on long-distance referrals. São Paulo concentrated 7,112 procedures. The spatial distribution of surgical volume reflected a marked inequality in capacity, particularly in the Amazon basin and the semi-arid Northeast.

In-hospital mortality varied substantially by location. Large-volume centers such as São Paulo and Belo Horizonte showed relatively controlled mortality, while several low-volume municipalities exhibited substantially elevated rates. The highest surgical mortality centers were Macapá (26.83%), Guarulhos (25.33%), Criciúma (23.08%), and Porto Velho (22.22%). This geographic variation is consistent with volume-outcome relationships described in high-income countries’ cardiac surgery registries and provides quantitative support for prioritizing surgical capacity expansion in underserved regions.

**Figure 1:**
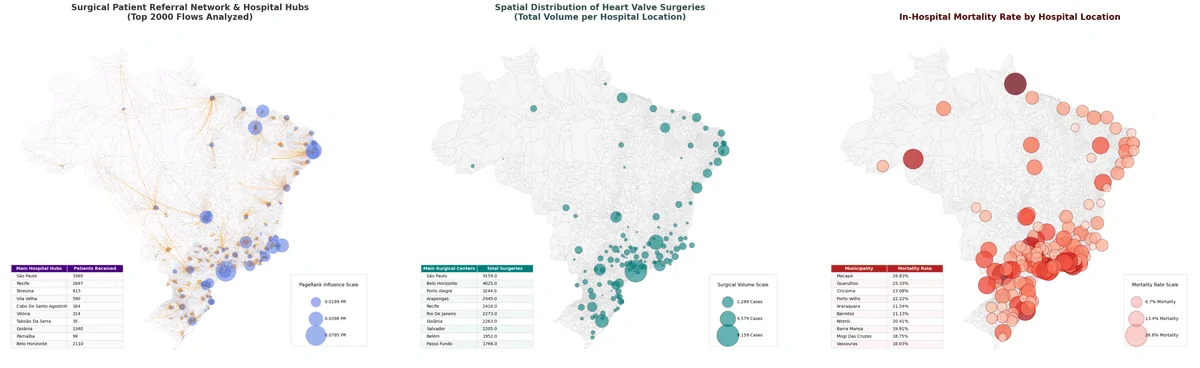
Spatial analysis of heart valve surgery in Brazil. Left: Surgical patient referral network and hospital hubs (top 2,000 flows; bubble size = PageRank influence). Center: Total volume of valve surgeries per hospital location. Right: Surgical in-hospital mortality rate by municipality.

Institutional-level analysis identified 250 hospitals performing valve surgery during the study period. High-volume centers (>200 cases/year) accounted for 102 hospitals and 51,152 patients, with an in-hospital mortality of 8.01%, whereas low-volume centers (≤200 cases/year) accounted for 148 hospitals and 12,274 patients, with a mortality of 11.05%. This institutional volume-outcome pattern paralleled the spatial distribution of surgical mortality described above (Figure 1), with low-volume centers disproportionately located in underserved regions.

### 3.4 Mortality outcomes by etiology and time horizon

Across the cohort (Table 2), the in-hospital, 30-day, 1-year, 5-year, and 10-year mortality rates were 8.6%, 10.0%, 19.6%, 30.1%, and 33.3%, respectively. These figures are substantially higher than benchmarks from high-income country registries: Siregar et al. reported 4.2% of 30-day mortality in the Netherlands (12,13), and Lee et al. documented 6.3% across more than 600,000 North American procedures (14). Leading causes of death included rheumatic mitral stenosis, myocardial infarction, and unspecified viral infection.

**Table 2:** Mortality outcomes by etiological groups across multiple time horizons.


ETIOLOGY_GROUP	TOTAL DEATHS (n)	OVERALL MORTALITY %	IN-HOSPITAL % (n)	30-DAY % (n)	1-YEAR % (n)	5-YEAR % (n)	TOP ICD-10 CAUSE

Overall	21130	33.3%	8.6% (5451)	10.0% (6331)	19.6% (12424)	30.1% (19108)	Mitral Stenosis

Degenerative	10331	33.7%	7.9% (2422)	9.4% (2887)	19.6% (6015)	30.5% (9329)	Aortic Stenosis

Rheumatic	8408	32.0%	8.5% (2243)	9.8% (2577)	18.4% (4845)	28.9% (7577)	Mitral Stenosis

Other	1683	37.5%	12.3% (553)	13.6% (610)	24.9% (1118)	34.7% (1559)	Endocarditis

Congenital	708	34.4%	11.3% (233)	12.5% (257)	21.7% (446)	31.3% (643)	Ebstein’s Anomaly


**Figure 2:**
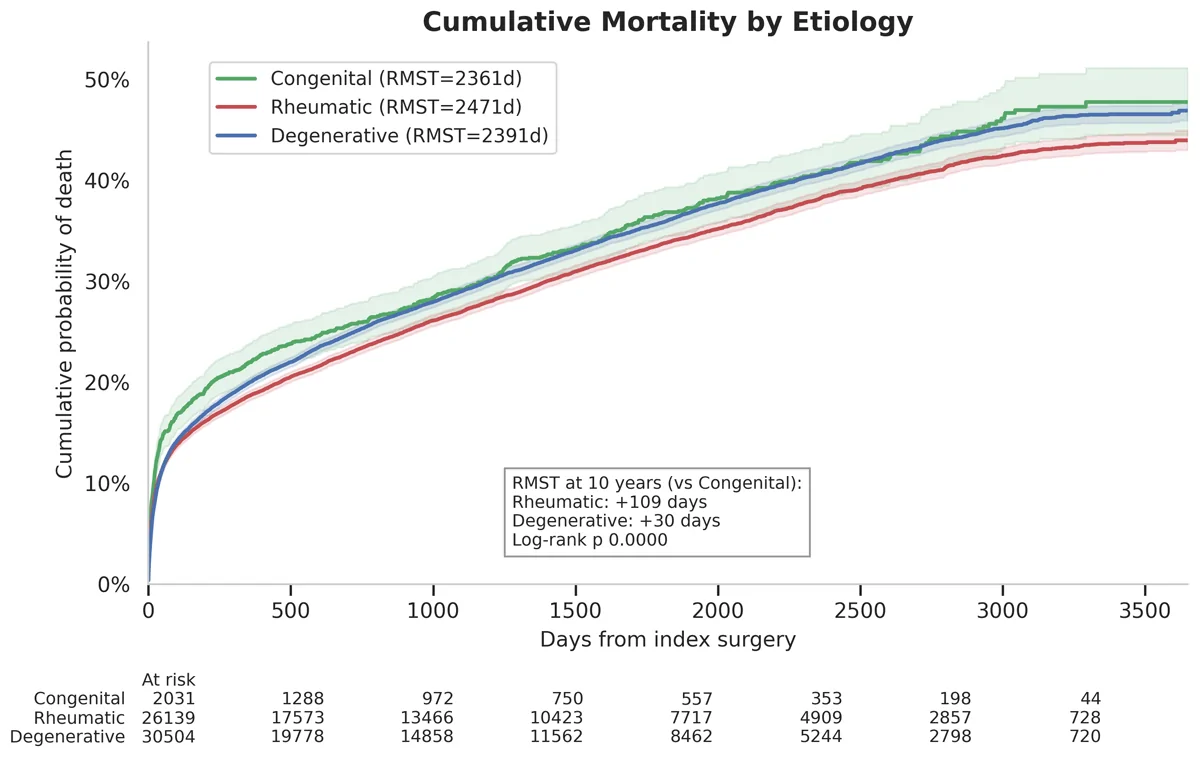
Kaplan–Meier curves for cumulative long-term mortality according to valve disease etiology, with restricted mean survival time (RMST) shown for each etiological group. Log-rank test: p < 0.001.

Etiology significantly predicted outcomes. Congenital disease had the highest 30-day mortality (12.5%), degenerative the lowest (9.4%), and rheumatic cases were intermediate (9.8%). Mixed or other etiologies carried the greatest long-term mortality risk. Median time to death ranged from 124 days in congenital to 204 days in degenerative patients (Figure 2).

### 3.5 Mortality outcomes by valve lesion subtype

**Figure 3:**
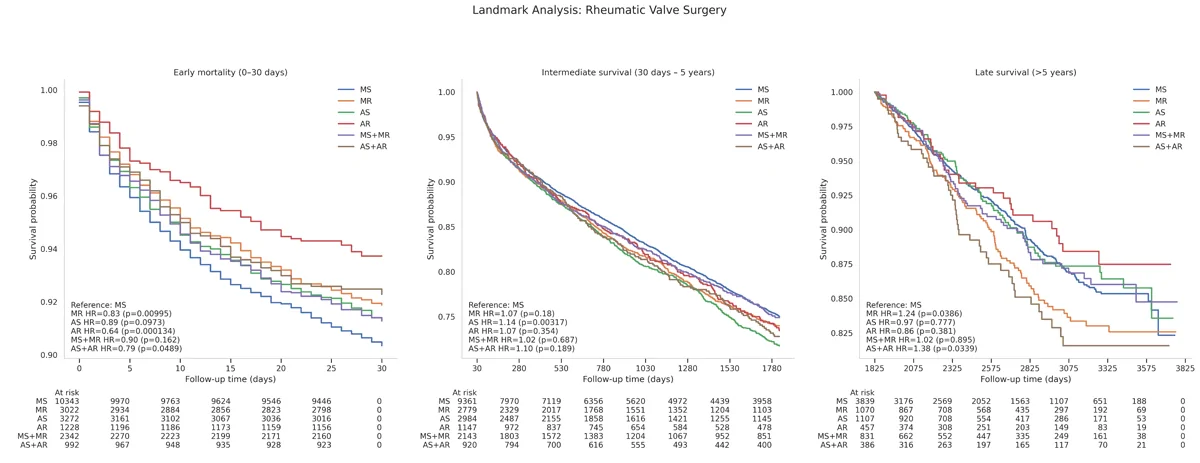
Kaplan–Meier survival curves by valve lesion subtype in the rheumatic group. Landmark analysis at 30 days and five years. Reference: Mitral stenosis. Log-rank p < 0.001.

Within the rheumatic group, (Figure 3) valve lesion subtype was significantly associated with survival after prosthetic replacement. Using mitral stenosis as reference, a lower 30-day mortality was observed for mitral regurgitation (HR 0.83; p = 0.010), aortic regurgitation (HR 0.64; p < 0.001), and combined aortic stenosis (AS) plus aortic regurgitation (AR) (HR 0.79; p = 0.049). Between 30 days and five years, aortic stenosis was the only subtype associated with higher mortality (HR 1.14; p = 0.003). Beyond five years, AS+AR remained associated with increased mortality (HR 1.38; p = 0.034), and mitral regurgitation emerged as an additional independent risk factor (HR 1.24; p = 0.039).

**Figure 4:**
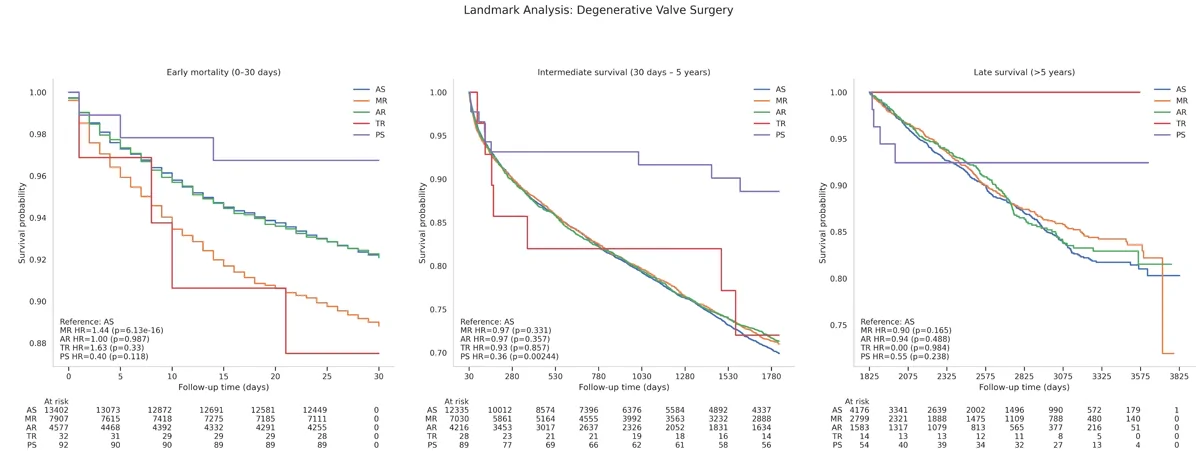
Kaplan–Meier survival curves by valve lesion subtype in the degenerative group.

**Figure 5:**
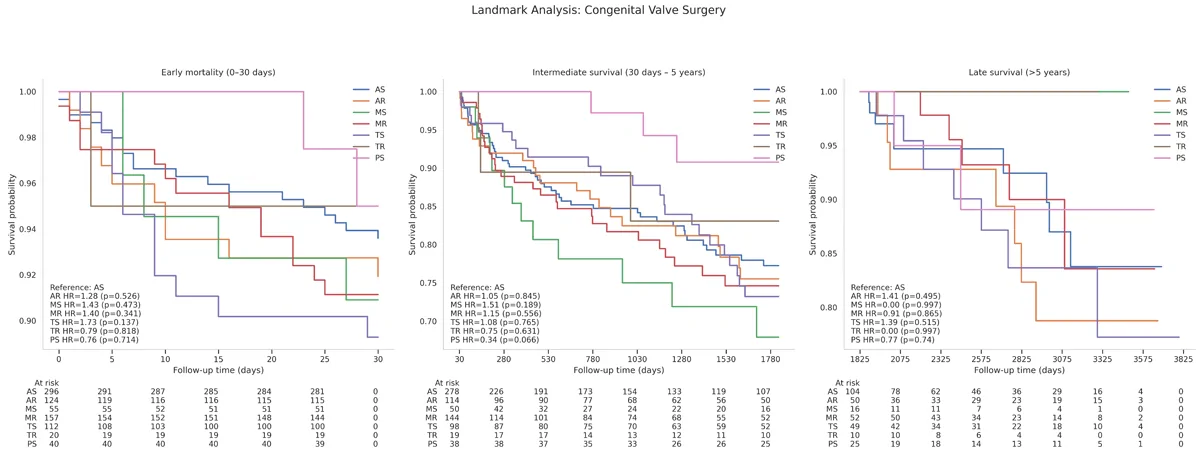
Kaplan–Meier survival curves by valve lesion subtype in the congenital group. Estimates beyond five years should be interpreted with caution given limited follow-up.

In the degenerative group (Figure 4), mitral regurgitation was associated with a higher 30-day mortality than aortic stenosis (HR 1.44; p < 0.001), with no significant differences at intermediate or late timepoints. Prosthetic valve disease was associated with substantially lower intermediate-term mortality (HR 0.36; p = 0.002), though this subgroup was small (n = 92) and the finding should be interpreted with caution. In the congenital group, (Figure 5) no significant differences were observed between lesion subtypes.

### 3.6 Survival by income quartile

To evaluate the independent contribution of socioeconomic status to survival, patients were stratified by residential income quartiles (Q1–Q4), and this stratification was entered as a covariate in the primary multivariable Cox model adjusted for age, sex, year of surgery, etiology, and region. Patients in the highest income quartile (Q4) had significantly lower mortality than those in the lowest quartile (Q1) in the adjusted Cox model (HR 0.82; 95% CI 0.76–0.89; p < 0.001). The corresponding adjusted Kaplan–Meier curve, stratified by income quartile, is presented in Supplemental Figure 2; survival curves diverged from the perioperative period onward, indicating that the income-associated mortality gap is not fully accounted for by differences in post-discharge follow-up intensity.

### 3.7 Reoperation: Incidence, temporal patterns, and mortality impact

Among 63,426 patients, 2,099 (3.31%) underwent at least one valve reoperation, with a mean inter-surgical interval of 3.1 ± 2.7 years. Crude mortality was higher in reoperation patients (38.0% overall; 15.2% at 30 days; 35.9% at 5 years) than in primary surgery patients (33.2%; 9.8%; 29.9%). Kaplan–Meier analysis confirmed a significantly worse survival trajectory for reoperation patients (HR 1.48; 95% CI 1.37–1.58; log-rank p < 0.001). In logistic regression, longer inter-surgical intervals were associated with lower in-hospital mortality (OR 0.89; 95% CI 0.86–0.93; p < 0.001). In Cox analysis, each additional year of inter-surgical interval was independently protective (HR 0.80; 95% CI 0.77–0.83; p < 0.001).

### 3.8 Post-surgical healthcare resource utilization

Post-operative care was characterized by high utilization of both general and cardiac-specific services, including physiotherapy (93,033 encounters), specialist consultations (72,170), and Doppler ultrasounds (61,788), alongside 28,105 transthoracic echocardiograms, 22,289 prothrombin time measurements, 21,536 electrocardiograms, and 6,606 Holter-monitoring studies. The most frequent diagnoses during follow-ups were unspecified heart failure (ICD-10: I50.9; n = 53,775; 15.1%), aortic valve stenosis (I35.0; 5.1%), mitral stenosis (I05.0; 3.7%), and mitral regurgitation (I34.0; 3.1%).

## 4. Discussion

This study analyzed outcomes in 63,426 patients undergoing prosthetic heart valve surgery within the SUS, representing the largest nationwide cohort reported from an LMIC and the first to link five national administrative databases to reconstruct individual patient trajectories from prior clinical history through extended follow-up. Its principal contribution lies in demonstrating, at a population scale, that post-operative mortality is substantially higher than in high-income country registries, that this excess mortality is spatially and socioeconomically patterned, and that specific structural and clinical factors, including institutional surgical volume and inter-surgical interval, are associated with survival. The high prevalence of rheumatic disease in this cohort (41.4%), approaching that of degenerative causes (48.3%), reflects its persistent epidemiological and social significance in middle-income settings and underscores the relevance of these findings beyond the Brazilian context.

The 30-day mortality of 10.0% in this cohort substantially exceeds benchmarks from the Netherlands (4.2%) and North America (6.3%) (12,13,14), and is also higher than the 7.3% reported by the BYPASS Registry from Brazil (9,15). This excess is likely multifactorial, encompassing the predominance of rheumatic disease with its associated pattern of delayed presentation and prolonged hemodynamic compromise (16), the structural limitations of a healthcare system in which cardiac surgical capacity remains concentrated in a small number of high-volume centers, and the burden of comorbidities characteristic of LMIC populations (17,18). Direct risk-adjusted comparisons with high-income registries are further constrained by the absence of functional status and EuroSCORE data in administrative records. Nonetheless, the magnitude of the difference is sufficiently large that case-mix alone is unlikely to account for it entirely. Notably, although the SUS serves a population more than 12 times larger than that of the Netherlands, the two systems perform a comparable absolute number of valve procedures annually, a disproportion that quantifies the scale of the unmet surgical need and its disproportionate impact on those with the least access to care.

The geographic distribution of surgical activity revealed a pattern that is structurally consistent with Brazil’s broader socioeconomic geography: the vast majority of high-volume centers are concentrated along the Atlantic coastline, particularly in the more economically developed Southeast and parts of the Northeast, while the interior and the entire North region—comprising the Amazon basin and neighboring states—are characterized by a near-complete absence of established cardiac surgical infrastructure. São Paulo emerged as the dominant national referral hub, receiving more than twice the number of referred patients of the second-largest center, and concentrated 7,112 procedures over the study period. This coastal concentration reflects historical patterns of urban and economic development that have systematically left the interior and the North with limited specialist healthcare capacity (19,20). Municipalities in these underserved regions rely on long-distance referrals that themselves represent a barrier to timely care, and the data show that this translates into measurable mortality consequences: Macapá and Porto Velho, capitals of states in the Far North, ranked among the four municipalities with the highest in-hospital mortality rates in the entire country. The geographic variation in mortality documented here is consistent with well-established volume-outcome relationships in cardiac surgery and provides a direct quantitative basis for prioritizing surgical capacity expansion in regions currently outside the reach of adequate care (21,22). Moreover, this pattern was corroborated at the institutional level, where high-volume centers (>200 cases/year) showed a mortality of 8.01% compared with 11.05% among low-volume centers, reinforcing surgical capacity as a modifiable structural factor underlying these geographic disparities.

Perioperative mortality across etiological groups was broadly consistent with the hemodynamic characteristics of the underlying valvular lesion. Within the rheumatic subgroup, aortic regurgitation was associated with lower early mortality than mitral stenosis (HR 0.64), a finding interpretable in the context of its more gradual hemodynamic progression and the consequently less acute physiological burden at the time of surgical intervention. In the degenerative group, mitral regurgitation was associated with higher perioperative mortality than aortic stenosis (HR 1.44), with no significant differences observed at intermediate or late timepoints. Of particular relevance for clinical management in LMIC populations is the time-varying risk structure identified in the rheumatic group. The late emergence of mitral regurgitation as an independent risk factor beyond five years (HR 1.24) and the persistent excess mortality associated with combined aortic stenosis and aortic regurgitation at the same time horizon (HR 1.38) have direct implications for postoperative surveillance and the timing of reintervention. These findings are particularly relevant in settings where current guidelines are largely extrapolated from high-income country series with different disease profiles.

Income was independently associated with surgical survival in the adjusted Cox model, retaining significance even after adjustment for etiology, region, and year of surgery. Patients in the highest income quartile had significantly lower mortality than those in the lowest (HR 0.82; 95% CI 0.76–0.89), with survival curves diverging from the perioperative period onward. This early divergence indicates that the income-associated mortality gap operates at least in part before discharge, suggesting that lower-income patients may present with more advanced disease, higher hemodynamic burden, and greater surgical risk at the time of referral.

Most cardiac surgical procedures in Brazil are performed within the SUS, and the annual surgical volume observed should be interpreted against a system characterized by a substantial waiting list for elective valve surgery rather than by low underlying demand. The relatively modest number of procedures per year (~6000 procedures/year) reflects a limited number of centers credentialed to perform valve replacement—a structural bottleneck—rather than a ceiling set by disease burden. This same imbalance helps explain the counterintuitive age distribution: despite substantially higher mortality than high-income country registries, mean age at surgery in this cohort was lower, a pattern largely driven by the high proportion of rheumatic etiology (41.4%), in which valvular damage begins in childhood or adolescence and progresses, under conditions of limited access to early diagnosis and longitudinal follow-up, in this vulnerable population. Furthermore, annual in-hospital mortality declined gradually from 9.46% in 2015 to 7.80% in 2020, coinciding with the pandemic-related 34% reduction in surgical volume, then rose transiently to 9.03% in 2021—potentially reflecting a backlog of more advanced diseases among postponed patients—before stabilizing between 7.95% and 8.34% from 2022 through 2024. Together, these findings reflect persistent socioeconomic and infrastructural inequity in access to cardiac surgical care, of which income, geography, and etiology are interrelated manifestations rather than independent findings.

Brazil is one among many middle-income countries facing the intersection of a persistent rheumatic disease burden, geographically unequal surgical infrastructure, and socioeconomic factors that systematically disadvantage the populations most in need of surgical care. The patterns documented in this paper, including the concentration of mortality in low-volume peripheral centers, the income gradient in perioperative survival, and the late excess risk associated with specific valve lesion subtypes in rheumatic populations, are not specific to Brazil. They reflect structural conditions common to LMICs across sub-Saharan Africa, South and Southeast Asia, and Latin America, where similar disparities in surgical access and socioeconomic vulnerability are likely to generate comparable outcome differentials (23,24,25,26), even if population-level data to quantify them remain largely absent. In this respect, the present study provides not only a description of inequity within a single health system, but a framework and a set of quantitative benchmarks against which other LMICs with administrative health data infrastructure could begin to measure their own performance.

## 5. Limitations

Several limitations should be acknowledged. First, although median follow-up was 1,023 days, 28.8% of patients lacked one full year of observation, preventing complete distinction between death, transition to private care, and administrative loss to follow-up. Second, long-term estimates in the congenital subgroup remain subject to substantial uncertainty due to residual loss to follow-up. Third, key clinical variables including echocardiographic data, prosthesis type, functional status, and surgeon volume were unavailable. Fourth, etiological classification relied on ICD-10 codes and is potentially subject to misclassification. Fifth, mortality may be underestimated for patients who die outside the SUS, particularly in the North and Northeast regions. Finally, because the SIH only captures procedures financed by the SUS, this cohort does not include procedures performed exclusively within the private or supplementary health sector, which may limit the generalizability of these findings to the Brazilian health system as a whole (see Data Availability Statement).

## 6. Conclusion

Valve-specific mortality patterns, reoperation risk, and income-based disparities are associated with post-operative survival outcomes after valve surgery in Brazil’s public health system. The socioeconomic and geographic inequities documented here are consistent with structural conditions shared across LMICs, where rheumatic disease persists as a prevalent cause of valve disease, and access to cardiac surgery remains severely limited. Brazil, as the world’s largest single-payer health system, provides both a quantitative benchmark and a replicable methodological framework for cardiovascular outcome surveillance in resource-constrained settings.

## Additional Files

The additional files for this article can be found as follows:

10.5334/gh.1581.s1 Supplemental Figure 1. Kaplan–Meier survival curves comparing primary surgery versus reoperation patients.

10.5334/gh.1581.s2 Supplemental Figure 2. Kaplan–Meier curves (adjusted Cox model) for survival by income quartile (Q4 vs. Q1). Q4 showed better survival (HR 0.82; 95% CI 0.76–0.89; p < 0.001).

## Data Availability

Data are available upon reasonable request, in accordance with the data access regulations for administrative databases established by the Brazilian Ministry of Health/DATASUS. The Hospital Information System (SIH) captures procedures financed by Brazil’s Unified Health System (SUS) but does not capture procedures performed exclusively within the private or supplementary health sector; this should be considered when interpreting the database.
