# Supplementary material for: Income, Geography, and Etiology as Factors Associated with Valve Replacement Outcomes: A Nationwide Cohort of 63,426 Patients in a Middle-Income Country
Source: Glob Heart. 2026 Aug 25;21(1):63. doi: 10.5334/gh.1581 (PMC13523802; doi:10.5334/gh.1581)
Supplement: Supplementary file 1 [file gh-21-1-1581-s1.pdf]

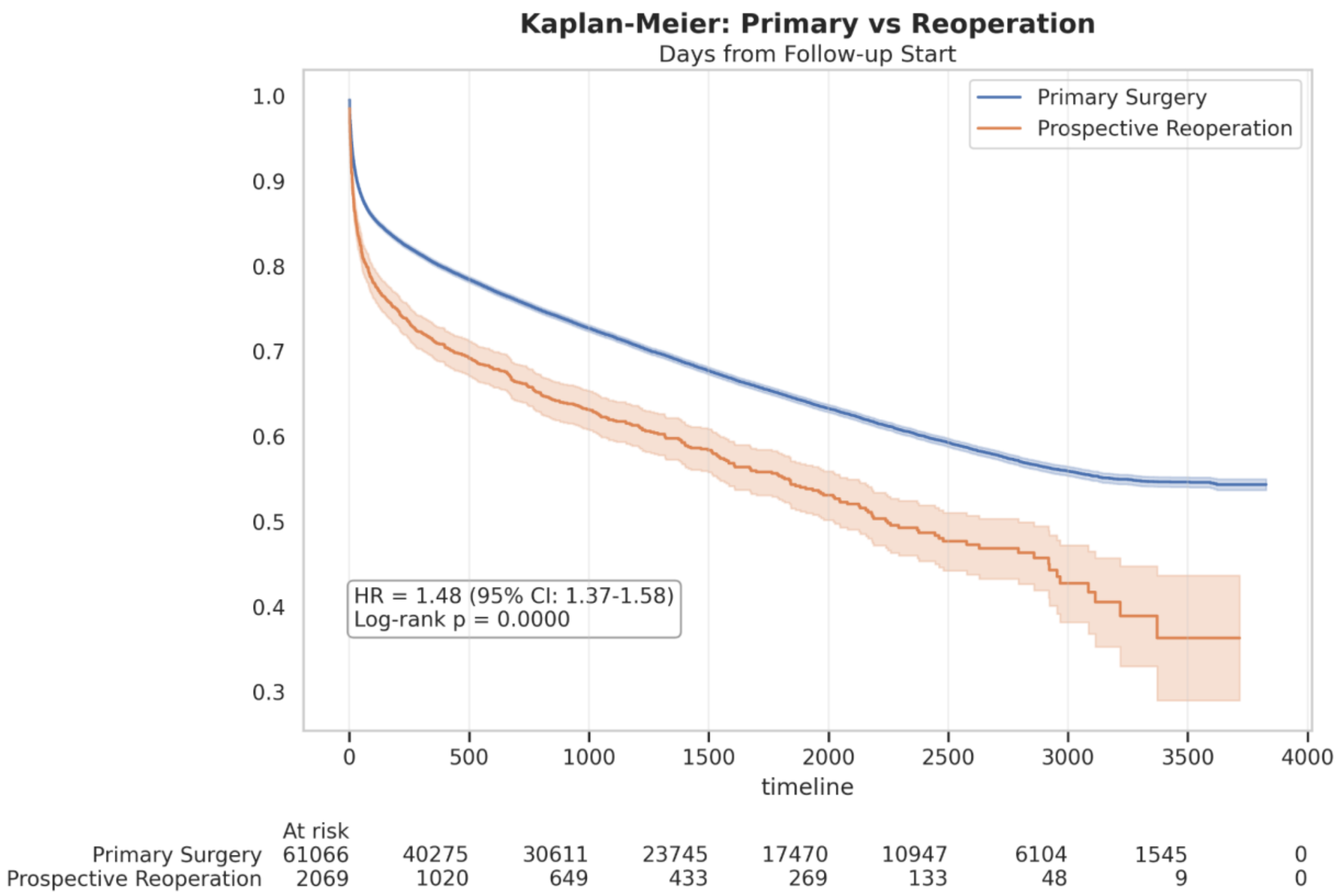

Supplemental Figure 1. Kaplan-Meier survival curves comparing time to death in primary surgery versus reoperation patients.
