# Supplementary material for: Income, Geography, and Etiology as Factors Associated with Valve Replacement Outcomes: A Nationwide Cohort of 63,426 Patients in a Middle-Income Country
Source: Glob Heart. 2026 Aug 25;21(1):63. doi: 10.5334/gh.1581 (PMC13523802; doi:10.5334/gh.1581)
Supplement: Supplementary file 2 [file gh-21-1-1581-s2.pdf]

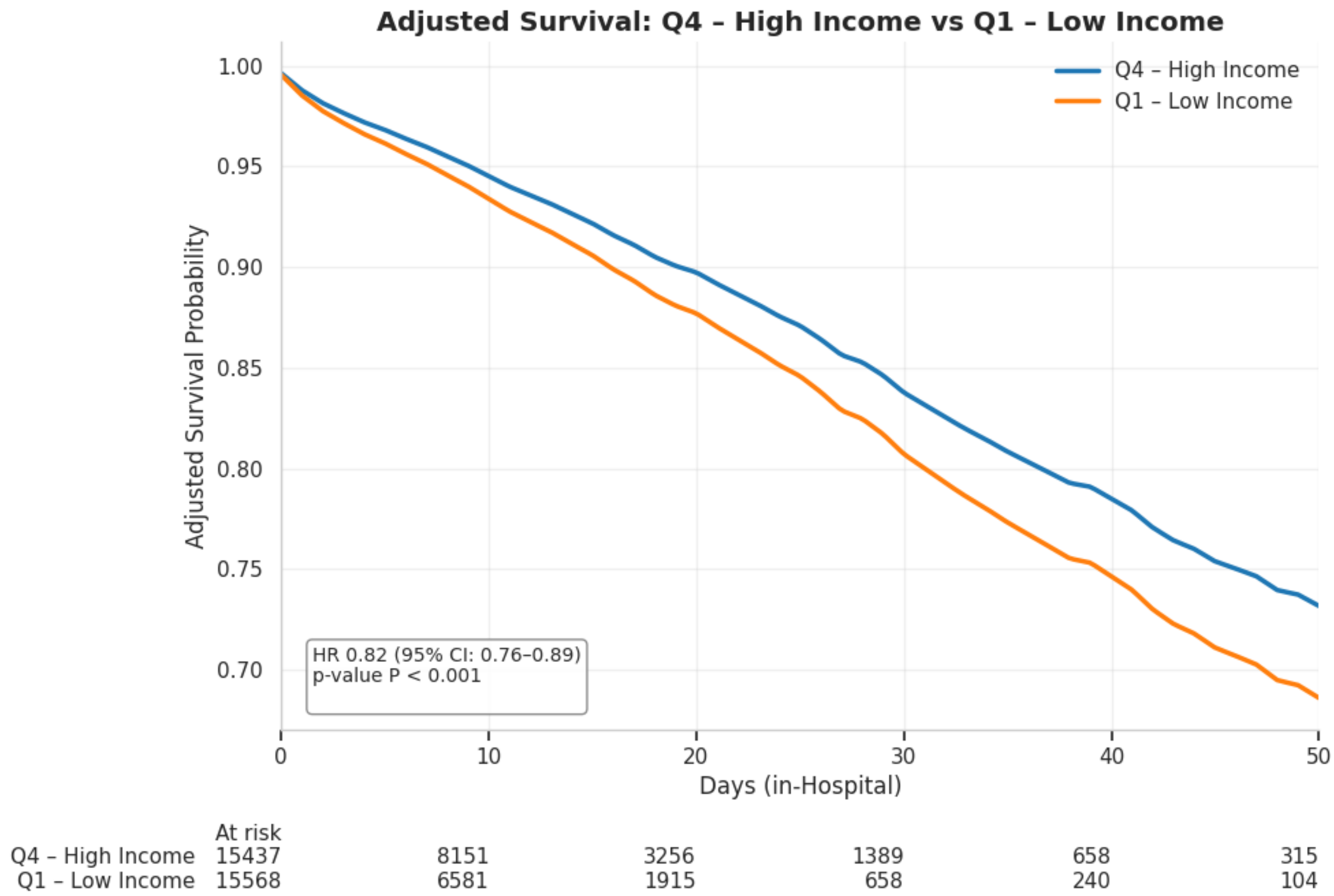

Supplemental Figure 2. Kaplan–Meier curves (adjusted Cox model) for survival by income quartile (Q4 vs Q1). Q4 showed better survival (HR 0.82; 95% CI 0.76–0.89; $p < 0.001$).
